# From trees to graphs: rethinking phylogeny in microbiome prediction

**DOI:** 10.1128/aem.01432-26

**Published:** 2026-09-01

**Authors:** Qiao Yu, Defeng Bai, Yao Wang, Yong-Xin Liu

**Affiliations:** 1 State Key Laboratory of Tropical Crop Breeding, Genome Analysis Laboratory of the Ministry of Agriculture and Rural Affairs, Agricultural Genomics Institute at Shenzhen, Chinese Academy of Agricultural Sciences 441191, Shenzhen, China; Michigan State University, East Lansing, Michigan, USA

**Keywords:** microbiome prediction, phylogeny, graph convolutional networks (GCNs)

## Abstract

Microbiome prediction models overlook phylogeny or fail to preserve evolutionary structure. In a recent *Applied and Environmental Microbiology* article (B. Dong, B. Wang, J. Chen, X. Xu, and Z. Z. Xu, Appl Environ Microbiol 92:e00788-26, 2026, https://doi.org/10.1128/aem.00788-26), Dong et al. present PhyloGCNE, a graph-convolutional framework that addresses these limitations by preserving evolutionary topology and learning adaptive edge-aware signal propagation. This commentary evaluates whether and when phylogenetic information improves microbiome-based prediction and considers how biological relevance and transferability can be established across contexts.

## COMMENTARY

Microbiome-based prediction is increasingly used to identify disease-associated signatures and classify host phenotypes, yet a central representational mismatch remains in how microbial evolutionary structure is incorporated. A recent study by Dong et al. investigates whether the phylogenetic structure of microbial communities can be directly integrated into predictive models rather than treated as background information (1). Microbiome community profiles are inherently sparse and high-dimensional, whereas cohort sizes are often limited relative to the number of measured taxa. Many established workflows therefore represent taxa as independent features in a feature matrix (2–5). This simplification overlooks the evolutionary relatedness among microbial taxa and the partial phylogenetic conservation of many microbial traits (6).

The field is beginning to move beyond taxon-based representation. Recently, phylogeny-aware methods have reordered features for convolution, projected phylogenetic distances into Euclidean embeddings, or aggregated signals through fixed tree operations (7–9). However, these phylogeny-aware methods are limited by simplified representations of evolutionary structure and fixed aggregation strategies, which may compromise phylogenetic topology, hierarchical dependencies, and task-specific lineage contributions.

**Fig 1:**
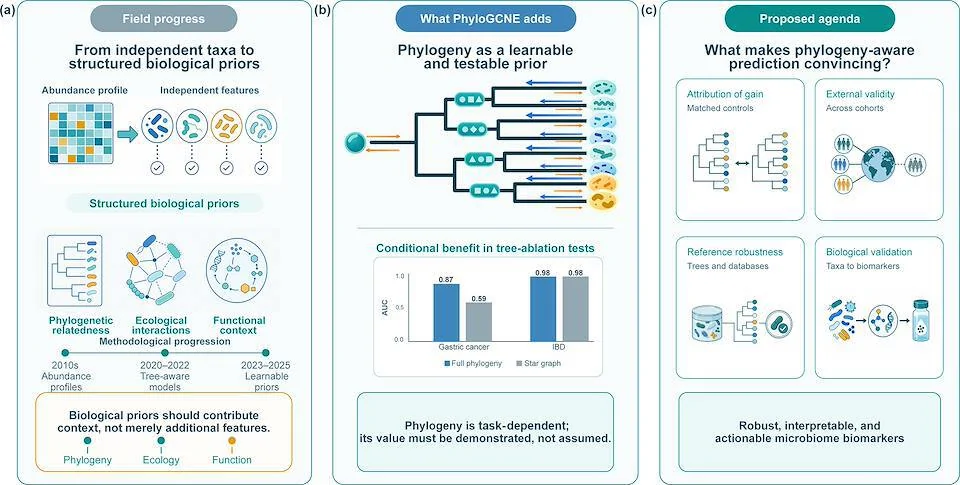
Phylogeny as a conditional, testable inductive bias in microbiome prediction. (**a**) Field progress from abundance profiles treated as independent features to phylogenetic, ecological, and functional biological priors. (**b**) PhyloGCNE represents a reference phylogeny as a graph and uses learnable edge-aware message passing to integrate hierarchical evolutionary information. Tree-ablation results illustrate its task-dependent benefit: median AUC decreased from 0.87 to 0.59 in gastric cancer when the full tree was replaced by a star graph, whereas both representations achieved 0.98 in IBD. (**c**) Four field-level priorities for credible phylogeny-aware prediction are attribution of gain, external validity, reference robustness, and biological validation. Quantitative values are derived from (1); the timeline and proposed agenda are synthesized in this commentary.

Dong et al. propose PhyloGCNE, which recasts the reference phylogenetic tree as a graph to enable adaptive learning of phylogeny-informed features (1, Fig. 1a). Observed microbial taxa are represented as leaf nodes, whereas internal nodes encode ancestral relationships and branch-derived edge features represent evolutionary proximity. Rather than reducing phylogeny to a feature ordering or distance embedding, PhyloGCNE learns how edge representations modulate message passing across the evolutionary hierarchy. The conceptual advance is therefore not merely a transition from trees to graphs but a shift from treating phylogeny as fixed background information to treating it as a learnable and testable biological prior. To improve interpretability, the authors further introduce Phylogenetic Saliency Propagation (PSP), which attributes model predictions to microbial taxa and higher-level lineages.

The authors benchmarked PhyloGCNE against DeepPhylo (8), Phylo-Spec (9), and random forest (RF) (10) using one synthetic data set and eight real-world cohorts representing 16S rRNA and shotgun metagenomic sequencing data. On the synthetic data set, PhyloGCNE achieved the highest median AUC (0.97) among the compared methods (1). Across the real-world cohorts, reported mean AUCs ranged from 0.73 to 0.99 (1). PhyloGCNE generally exceeded RF and DeepPhylo and was comparable to, or modestly better than, Phylo-Spec (1, 8–10). In the multiclass classification task, Phylo-Spec had a slightly higher median macro-averaged one-versus-rest AUC than PhyloGCNE (approximately 0.94 versus 0.92), whereas both methods achieved a Macro-F1 of 0.66. PhyloGCNE nevertheless showed more balanced recall across disease and control classes. Together, these comparisons motivate a closer examination of when the phylogenetic representation itself contributes to prediction.

The comparison between the full phylogenetic tree and a star graph representation tests whether the full phylogenetic hierarchy contributes to model performance (Fig. 1b). In the gastric cancer cohort, replacing the full tree with a star graph lowered the median AUC from approximately 0.87 to 0.59. In contrast, both graph representations achieved approximately 0.98 in the IBD cohort (1, Fig. 1b). This contrast supports treating phylogeny as a task-dependent inductive bias rather than as a uniformly beneficial feature of host phenotype prediction. However, the ablation does not isolate the effect of evolutionary relatedness. Replacing the tree with a star graph also removes multiscale hierarchical message passing. The gastric cancer results therefore support the importance of phylogenetic structure for prediction, but they do not establish whether the observed performance gain arises uniquely from evolutionary relatedness or from the broader benefits of hierarchical information aggregation.

Phylogenetic Saliency Propagation (PSP), the study’s interpretability framework, combines abundance-constrained SmoothGrad with top-down propagation of saliency scores along the phylogeny (1, 11) and represents another notable contribution of PhyloGCNE. By integrating phylogeny-aware saliency propagation with an independent feature elimination strategy, the authors move beyond conventional feature attribution and provide evidence that the highest-ranked taxa capture a substantial proportion of the predictive signal across multiple cohorts. Importantly, validating the selected features with an independent random forest classifier (10) reduces concerns that the observed importance scores merely reflect model-specific behavior. Nevertheless, these results should be interpreted as evidence of predictive relevance rather than biological causality. Saliency scores remain sensitive to feature correlation, compositional constraints, and the choice of reference phylogeny (12), while the identified taxa may represent lineage-level biomarkers or ecological proxies rather than direct disease drivers (13–15). Future studies integrating longitudinal cohorts and experimental validation will be essential to determine whether these phylogeny-informed biomarkers reflect causal mechanisms or simply stable correlates of host phenotype.

Although PhyloGCNE demonstrates strong predictive performance and improved interpretability, opportunities remain for further methodological refinement and broader validation. Incorporating uncertainty estimation and probability calibration would improve the reliability of model predictions in potential clinical applications (16). Integrating phylogenetic graphs with complementary biological representations, including gene-sharing, metabolic, and ecological interaction networks, could help capture processes that are not fully explained by vertical inheritance alone, such as horizontal gene transfer and cross-lineage microbial interactions (17). More broadly, because the predictive value of microbial phylogeny appears to vary across diseases, future microbiome-based host phenotype prediction models may benefit from adaptively integrating disease-specific phylogenetic information with complementary functional, ecological, and host-derived features.

An equally important next step is to disentangle the contribution of phylogenetic relatedness from other properties of hierarchical graph models. Analyses across alternative reference phylogenies, including the updated Greengenes2 resource (18), should establish whether conclusions depend on the resource used. A shuffled-tree control that preserves topology and branch-length distributions while permuting leaf labels would adapt tip-label permutation null models used to test phylogenetic signal (19). It could determine whether the observed gains depend on biologically meaningful evolutionary neighborhoods rather than on graph structure alone. PhyloGCNE should also be compared with conventional classifiers trained on phylogenetically derived hierarchical abundance features or balances, approaches established in microbial community classification and compositional microbiome analysis (20, 21). This comparison would clarify whether gains arise from learned phylogenetic message passing rather than from multiscale aggregation alone. Finally, architecture-matched control models should separate the contributions of phylogeny, hierarchy, and model complexity. These are proposals of this commentary, rather than analyses already performed in the focal study (Fig. 1c).

Future work should also establish whether phylogeny-aware prediction generalizes across independent cohorts that differ in population characteristics, sequencing platforms, analytical workflows, body sites, and reference phylogenies. PhyloGCNE’s leave-one-dataset-out evaluation across colorectal cancer cohorts provides an encouraging first step toward assessing generalizability, but broader validation under realistic sources of domain shift will be essential for determining the robustness and transferability of phylogeny-aware prediction (1, 22). Equally important, translating model interpretation into biological insight remains a major challenge. Candidate taxa identified by PhyloGCNE should therefore be evaluated for consistency across training splits, independent cohorts, taxonomic databases, and alternative phylogenetic reconstructions before they can be considered robust biomarkers (13–15). More broadly, phylogeny represents only one dimension of the microbial community. Horizontal gene transfer can decouple functional traits from species ancestry (17), whereas ecological interactions and metabolic dependencies frequently connect evolutionarily distant taxa. Integrating evolutionary, functional, and ecological information therefore represents a promising direction for future phylogeny-aware microbiome prediction.
